# Photoactivation of hypericin decreases the viability of RINm5F insulinoma cells through reduction in JNK/ERK phosphorylation and elevation of caspase-9/caspase-3 cleavage and Bax-to-Bcl-2 ratio

**DOI:** 10.1042/BSR20150028

**Published:** 2015-05-27

**Authors:** Jingwen Yi, Xiaoguang Yang, Lihua Zheng, Guang Yang, Luguo Sun, Yongli Bao, Yin Wu, Yanxin Huang, Chunlei Yu, Shao-Nian Yang, Yuxin Li

**Affiliations:** *National Engineering Laboratory for Druggable Gene and Protein Screening, Northeast Normal University, Changchun 130024, China; †Jilin Academy of Traditional Chinese Medicine, Changchun 130012, China; ‡School of Chemistry, Northeast Normal University, Changchun 130024, China; §The Rolf Luft Research Center for Diabetes and Endocrinology, Karolinska Institutet, SE-171 76 Stockholm, Sweden

**Keywords:** anti-cancer, cell destruction, cell growth, hypericum perforatum, pancreatic endocrine tumour, photopharmacology, Bcl-2, B-cell lymphoma 2, ERK, extracellular-signal-regulated kinase, GABA, γ-aminobutyric acid, GAPDH, glyceraldehyde 3-phosphate dehydrogenase, 5-HT_1_, 5-hydroxytryptamine_1_, JNK, c-Jun N-terminal kinase, LED, light-emitting diode, MAPK, mitogen-activated protein kinase, NF-κB, nuclear factor κ-light-chain-enhancer of activated B cells, PMA, phorbol-12-myristate-13-acetate, ROS, reactive oxygen species, TNF-α, tumor necrosis factor α

## Abstract

Insulinomas cause neuroglycopenic symptoms, permanent neurological damage and even death. Current available therapies cannot satisfactorily treat malignant insulinomas and some benign insulinomas. The promising phototherapeutic results and harmless side effects of hypericin in some cancer treatments prompted us to explore possible anti-growth activity of photoactivated hypericin against RINm5F insulinoma cells and underlying mechanisms. We now show that detectable and maximal internalization of hypericin in RINm5F insulinoma cells occurred in 20 and 60 min respectively. Hypericin was considerably associated with the plasma membrane, appreciably localized in the sub-plasma membrane region and substantially accumulated in the cytoplasm. Photoactivated hypericin decreased the viability of RINm5F insulinoma cells due to its anti-proliferative and apoptotic actions. Photoactivation of hypericin inhibited cell proliferation reflected by decreased expression of the proliferation marker Ki-67 and cell-cycle arrest in the G_0_/G_1_-phase. The anti-proliferative effect resulted from down-regulation of phosphorylation of c-Jun N-terminal kinase (JNK) and extracellular-signal-regulated kinase (ERK). Photoactivated hypericin triggered apoptosis through activation of caspase-3 and caspase-9 and elevation of the Bax-to B-cell lymphoma 2 (Bcl-2) ratio. The findings lay a solid foundation for implementation of hypericin-mediated photodynamic therapy in treatment of insulinomas.

## Abstract 2

Photoexcited hypericin exerts strong antigrowth activity in RINm5F insulinoma cells via inhibition of cell proliferation by reducing JNK/ERK phosphorylation and stimulation of apoptosis by elevating caspase-9/caspase-3 cleavage and Bax-to-Bcl-2 ratio.

## INTRODUCTION

The perennial herb *Hypericum perforatum L.*, commonly known as St. John's wort, has long been used as a medicinal herb [1,2]. It bears a broad spectrum of pharmacological activities, e.g. anti-depressive, antiviral, anti-inflammatory and anti-tumoural effects [1,2]. These pharmacological actions occur due to various types of biologically active components in extracts of the herb. Both clinical and experimental studies verify that the red-coloured pigment hypericin is one of the principal active components responsible for the therapeutic effects [1,2]. Hypericin acts as an anti-depressant via multiple mechanisms such as γ-aminobutyric acid_A_ (GABA_A_) receptor and 5-hydroxytryptamine_1_ (5-HT_1_) receptor binding, inhibition of dopamine-β-hydroxylase and suppression of glutamate release [1,3]. This natural compound serves as a virucidal agent by reducing viral infectivity and replication [1,4]. Hypericin is also a multifaceted player in cell signalling [1,5–8]. For example, it abrogates the phorbol-12-myristate-13-acetate (PMA)- and tumor necrosis factor α (TNF-α)-induced activation of nuclear factor κ-light-chain-enhancer of activated B cells (NF-κB), inhibits epidermal growth factor receptor tyrosine kinase, insulin receptors and protein kinase C and reduces synthesis of prostaglandin-E2 and interleukin 6 [1,5–8]. This underlies the anti-inflammatory and anti-tumoural activities of hypericin [1,7].

Interestingly, the therapeutic effects of hypericin can be boosted over 100-fold in the presence of light [4]. This happens due to photochemical features of hypericin [4]. This plant pigment is presumably biosynthesized from its precursor emodin anthrone [1,2]. Chemically, hypericin is an aromatic polycyclic dione with a molecular mass of 504.4 Da and belongs to the naphthodianthrone family [4]. The aromatic rings of hypericin confer photochemical properties on this plant pigment, which has a broad absorbance spectrum with maxima at 545 and 595 nm and a wide range of emission wavelengths peaking at 594 and 640 nm [9,10]. Hypericin molecule harbours extended π-orbital electrons [1]. Upon illumination, the π-orbital electrons become excited to generate toxic singlet oxygen and radical species [1]. These unstable reactive intermediates cause oxidation of cell membrane lipids and cellular proteins in milliseconds [1,11]. Photo-excited hypericin can also specifically target purine nucleotides in DNA [12]. These phototoxicities result in cytostasis, mitochondrial damage, apoptosis and necrosis via complex mechanisms, such as reduction in ATP synthesis, inhibition of protein kinases, abrogation of cell growth signal transduction and activation of cell death signalling pathways [1,6,13–18]. Hypericin is a potent photosensitizer and produces the aforementioned effects at nanomolar levels [1,6,13–18]. In addition, non-photoactivated hypericin exhibits harmless side effects and preferentially accumulate in tumour cells [1,19–23]. These properties have made hypericin one of the most useful photosensitizers for photodynamic therapy of tumours [1,19–23]. Importantly, promising results of hypericin-mediated photodynamic application have been obtained in the diagnosis and treatment of skin, nasopharyngeal, bladder and pancreatic cancers as well as lymphomas, radiation-induced fibrosarcoma in patients or animal models [22–27].

Insulinomas are the most common, functioning, pancreatic neuroendocrine tumours [28–30]. They are derived from β-cells and over-secrete insulin resulting in hypoglycaemia [28–30]. Therefore, insulinoma patients present hypoglycaemia and neuroglycopenic symptoms, e.g. recurrent headache, lethargy, diplopia and blurred vision, seizures, coma and even permanent neurological damage [29,30]. Insulinomas can be well-differentiated, poorly-differentiated and undifferentiated respectively. If patients develop distant metastases, they have a median survival of less than 2 years [28–31]. Indeed, well-differentiated localized benign insulinomas can be cured by surgery [28–31]. However, surgery is contraindicated in patients with multiple benign insulinoma, unresectable malignant insulinoma and metastatic insulinoma as well as those with serious mental and physical problems [28–31]. Although these patients can receive chemotherapy and radiotherapy, these therapies produce unsatisfactory results and serious side effects [28–31]. The potent photodynamic activity, tumour cell-selective accumulation, promising phototherapeutic results and harmless side effects of hypericin in some cancer treatments prompted us to explore possible antigrowth activity of photoactivated hypericin against RINm5F insulinoma cells and underlying mechanisms [19,22–26]. In the present work, we demonstrate that photoactivation of intracellular hypericin hampers proliferation and triggers apoptosis of RINm5F insulinoma cells. This lays a solid foundation for implementation of hypericin-mediated photodynamic therapy in treatment of insulinomas.

## MATERIALS AND METHODS

### Cell culture

RINm5F insulinoma cells at ~70% confluency were trypsinized. The resultant suspension of cells was seeded into microplate wells or on to glass coverslips. The cells were cultivated in RPMI 1640 medium supplemented with 10% FBS (Biochrom), 2 mM L-glutamine and 100 units/100 μg/ml penicillin/streptomycin (Invitrogen) and maintained at 37°C in a humidified 5% CO_2_ incubator [32,33]. They were grown overnight and then subjected to different treatments.

### Live-cell confocal microscopy

Live-cell confocal microscopy was used to characterize the dynamics of hypericin internalization in RINm5F insulinoma cells. The cells were bathed in extracellular solution (pH 7.4) consisting of 135 mM NaCl, 3.6 mM KCl, 5 mM NaHCO_3_, 0.5 mM NaH_2_PO_4_, 0.5 mM MgCl_2_, 1.5 mM CaCl_2_, 10 HEPES and 0.1% BSA. Subsequent administration of hypericin at a concentration of 100 nM proceeded. Internalized hypericin in the cells was visualized with a Leica TCS-SP2 confocal laser-scanner connected to a Leica DMIRBE microscope (Leica Microsystems Heidelberg GmbH). A 488 nm laser line was used to excite hypericin and a Leica PL APO 100×/1.40 oil objective was employed to capture the resultant emission at 540–650 nm. Quantification of intracellular hypericin fluorescence intensity was performed with Leica Confocal Software (Leica). Live-cell confocal imaging of hypericin was conducted at 37°C.

### Photoactivation of intracellular hypericin

RINm5F insulinoma cells were plated in microplate wells with and without glass coverslips at a density of 1 × 10^4^/well and were grown overnight. Stably-attached cells were incubated for 4 h with hypericin at concentrations ranging from 12.5 to 200 nM and rinsed three times. The hypericin-treated cells were then illuminated for 10 min with a white light-emitting diode (LED) light source (intensity: 1.05 mW/cm^2^, wavelength: 470–625 nm). All the aforementioned steps were done at 37°C. Thereafter, they were cultured for 24 h, 2 h and 20 min respectively and subjected to MTT (Sigma–Aldrich) assay, immunofluorescence labelling, flow cytometry, DAPI staining and immunoblot analysis.

### MTT assay

Briefly, MTT (Sigma–Aldrich) was dissolved in PBS and filtered to make a 5 μg/ml MTT solution. 20 μl of the MTT solution was added to all the wells containing hypericin-treated cells subjected to photoactivation and kept at 37°C for 4 h. Subsequently, the solution was discarded and replaced with 100 μl/well of DMSO and followed by gentle shaking for 10 min. Finally, the microplates were read on a microplate reader (Bio-Rad Laboratories) at 570 nm. Experiments were performed in triplicate and the relative cell viability was expressed as a percentage relative to cells subjected to solvent treatment as control with or without photoactivation.

### Immunofluorescence labelling, DAPI staining and fluorescence microscopy

Immunofluorescence labelling was performed on RINm5F insulinoma cells following hypericin loading and photoactivation. The treated cells were then cultivated for 24 h. Thereafter, they were fixed with 4% paraformaldehyde for 30 min, permeabilized with 0.2% Triton X-100 for 5 min and blocked with 5% BSA for 30 min. The specimens were labelled with mouse monoclonal antibody against Ki-67 (1:500; Santa Cruz Biotechnology) at room temperature for 2 h. A subsequent incubation of the specimens with goat anti-mouse IgG coupled to Cy3 (1:500; Beyotime) proceeded for 30 min at room temperature. Afterwards, the specimens were incubated with a DAPI solution (5 μg/ml; Beyotime) at room temperature for 15 min. The specimens were mounted in an anti-fading medium and visualized with an inverted fluorescence microscope (Olympus IX-71). Their digital images were acquired with Olympus DP72 digital camera and analysed using Olympus DP2-BSW software.

### Flow cytometric analysis of apoptosis and cell cycle

After 50 nM hypericin exposure, photoactivation and cultivation, RINm5F insulinoma cells were stained with Annexin V-FITC/Propidium Iodide for detection of early apoptosis or propidium iodide for discrimination of cell cycle phases. Cells in the early phase of apoptosis and those in the G_0_/G_1_, S and G_2_/M phases of the cell cycle were quantified on a BD FACS Canto flow cytometer (Becton, Dickinson and Company).

### SDS/PAGE and immunoblot analysis

After hypericin loading, photoactivation and additional 20 min or 2 h culture, RINm5F insulinoma cells were lysed in a lysis buffer (pH 7.5) consisting of 20 mM HEPES-NaOH, 1 mM EDTA, 1 mM DTT, 20 mM NaF, 0.4 mM PMSF, 2 μg/ml pepstatin, 2 μg/ml aprotinin, 2 μg/ml leupeptin (Bioshop). The lysate was centrifuged at 800 ***g*** for 10 min at 4°C to remove cell debris and nuclei. The protein concentration of the resulting samples was determined with BCA protein assay reagent (Beyotime). The samples were denatured by heating at 100°C for 10 min in SDS sample buffer and then underwent SDS/PAGE and immunoblot analysis. Briefly, 30 μg of protein was separated in discontinuous gels consisting of a 5% acrylamide stacking gel (pH 6.8) and a 12% acrylamide separating gel (pH 8.8). The separated proteins were then electroblotted to PVDF membrane (Pierce). The blots were blocked by incubation for 1 h with 5% non-fat milk powder in a washing buffer, containing 20 mM tris(hydroxymethyl)aminomethane, 500 mM NaCl and 0.05% Tween 20 (pH 7.4). They were then incubated with different antibodies respectively, at 4°C for 12 h. These antibodies are listed below: mouse monoclonal antibodies to B-cell lymphoma 2 (Bcl-2) (1:500; Santa Cruz Biotechnology), glyceraldehyde 3-phosphate dehydrogenase (GAPDH) (1:10000; Kangcheng Biotech), c-Jun N-terminal kinase (JNK) (1:500; Cell Signaling Technology, Danvers, MA) and p38 (1:1000; Santa Cruz Biotechnology), respectively, as well as rabbit polyclonal antibodies to Bax (1:500; Santa Cruz Biotechnology), cleaved caspase-3 (1:1000; Cell Signaling Technology), cleaved caspase-9 (1:1000; Cell Signaling Technology), extracellular-signal-regulated kinase (ERK) (1:1000; Cell Signaling Technology), phospho-ERK (1:1000; Cell Signaling Technology), phospho-JNK (1:1000; Cell Signaling Technology), phospho-p38 (1:1000; Cell Signaling Technology) respectively. After rinsing with the washing buffer, the blots were incubated with the secondary antibodies (either horseradish peroxidase-conjugated goat anti-rabbit IgG or horseradish peroxidase-conjugated goat anti-mouse IgG; 1:2000; Dingguo Biotechnology) at room temperature for 45 min. The immunoreactive bands were visualized with Pierce ECL Western Blotting Substrate (Thermo Scientific).

### Statistical analysis

Data are presented as mean ± S.E.M. The statistical significance of differences between multiple groups was assessed by one-way ANOVA, followed by least significant difference (LSD) test. The statistical difference between two groups was determined by unpaired Student's *t* test. The significance level was set to 0.05 or 0.01.

## RESULTS

### Hypericin is internalized and accumulates in RINm5F insulinoma cells

**Figure 1:**
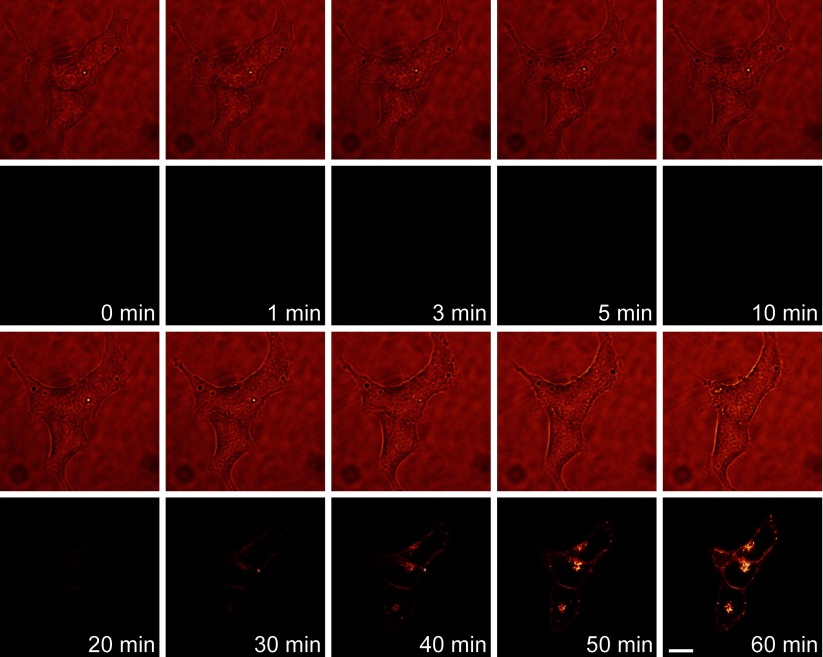
The cellular pharmacokinetic profile of hypericin in RINm5F insulinoma cells Representative live-cell confocal images (rows 2 and 4) and corresponding transmission images (rows 1 and 3) were acquired at indicated time points from cells exposed to 100 nM hypericin. Hypericin fluorescence became detectable in the plasma membrane and sub-plasma membrane region within 20 min and then appeared in the cytoplasm. Hypericin fluorescence in cells reached its maximum brightness in ~1 h. The experiments were repeated six times. Bar=10 μm.

The cellular pharmacokinetic profile of hypericin is the key prerequisite for characterizing photodynamic action of hypericin on the viability of RINm5F insulinoma cells. Therefore, we first visualized the real-time internalization and distribution of hypericin in RINm5F insulinoma cells using live-cell confocal microscopy. Figure 1 shows that extracellular hypericin at a concentration of 100 nM was efficiently internalized into cells within 1 h. Hypericin fluorescence was first visualized in the plasma membrane and sub-plasma membrane region within 20 min. Subsequently, it appeared in the cytoplasm (Figure 1). Obviously, hypericin not only bound to the plasma membrane, but also accumulated in the cytoplasm. Furthermore, the intensity of hypericin fluorescence in cells reached its maximum level in ~1 h. The uptake kinetics of hypericin in RINm5F insulinoma cells provides important guidelines for determining the optimal time point for photoactivation of intracellular hypericin. The subcellular accumulation pattern of hypericin in RINm5F insulinoma cells offers mechanistic hints for hypericin-mediated photodynamic action in these tumour cells.

### Photoactivated hypericin reduces the viability of RINm5F insulinoma cells

**Figure 2:**
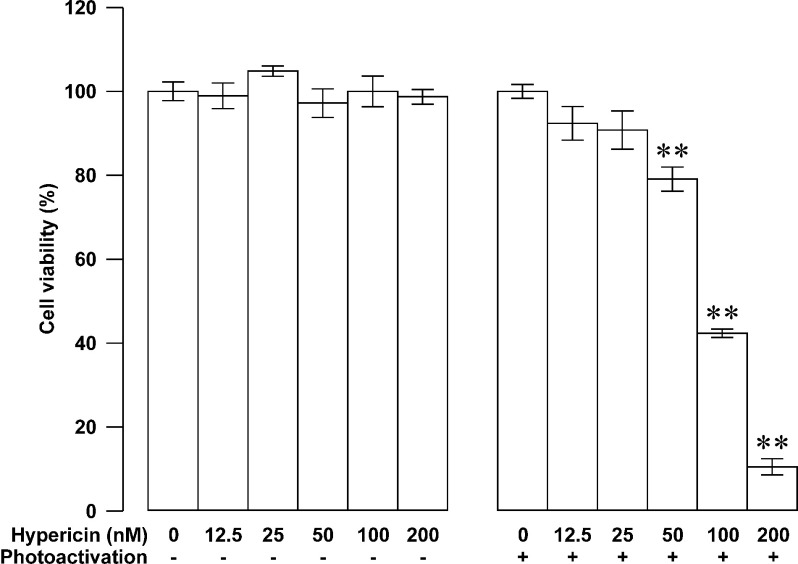
Photoactivated hypericin-induced reduction in the viability of RINm5F insulinoma cells The percentage of metabolically viable cells did not alter in groups loaded with indicated concentrations of hypericin without photoactivation (*N*=3; left panel). The percentage of metabolically viable cells decreased in groups treated with indicated concentrations of hypericin followed by photoactivation (*N*=3; right panel). Cell viability was measured using MTT assay. **P<0.01 compared with solvent control group exposed to 10-min photoactivation.

The first and foremost step in the process of developing an anticancer therapy is to assess its inhibitory ability against the viability of cancer cells. To explore the potential application of hypericin-photodynamic therapy in insulinomas, we performed MTT assay in RINm5F insulinoma cells loaded with hypericin followed by photoactivation. The treated cells were further grown for 24 h. In the concentration range of 12.5–200 nM, hypericin concentration-dependently inhibited the viability of RINm5F insulinoma cells following 10-min photoactivation. The percentage of metabolically viable cells decreased with elevation in hypericin concentration. The effect became statistically significant when hypericin concentration reached 50 nM and higher (*N*=3, *P*<0.01 at 50, 100 and 200 nM; Figure 2, right panel). The IC_50_ concentration was estimated to be 105.97 nM. In contrast, hypericin at the same concentrations had no effect on the percentage of metabolically viable RINm5F insulinoma cells without photoactivation. There was no significant difference in cell viability between hypericin-untreated (*N*=3) and hypericin-treated groups (*N*=3, *P*>0.05; Figure 2, left panel) in the absence of photoactivation. The data clearly show that photoactivation of hypericin potently reduces the viability of RINm5F insulinoma cells.

### Photoactivated hypericin inhibits RINm5F insulinoma cell proliferation through down-regulation of phospho-JNK and phospho-ERK

**Figure 3:**
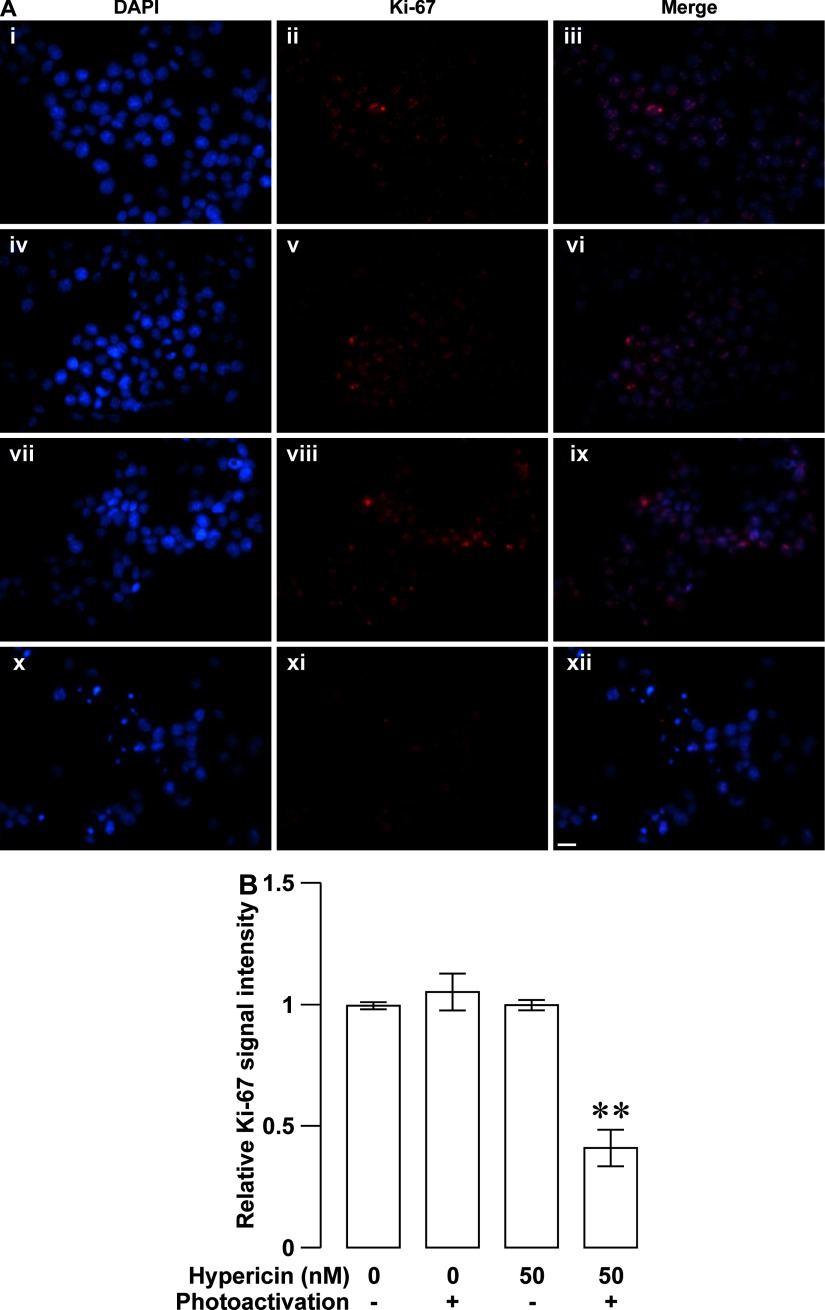
Photoactivated hypericin-induced down-regulation of the proliferation marker Ki-67 in RINm5F insulinoma cells (**A**) Sample Ki-67 immunofluorescence and DAPI fluorescence images were obtained in specimens subjected to solvent control treatment (control), hypericin loading (hypericin), solvent control treatment followed by photoactivation (control/photoactivation) and hypericin loading followed by photoactivation (hypericin/photoactivation). The majority of control- (**ii**), hypericin- (**v**), control/photoactivation-treated cells (**viii**) exhibited a similar intensity of Ki-67 immunoreactivity. A small proportion of hypericin/photoactivation-treated cells (**xi**) gave relatively weak Ki-67 immunoreactivity. Nuclei were verified by DAPI staining (**i**, **iv**, **vii** and **x**). Ki-67 immunoreactivity was exclusively visualized in nuclei marked with bright DAPI fluorescence (**iii**, **vi**, **ix** and **xii**). (**B**) Quantifications of the relative intensity of Ki-67 immunoreactivity. Control (*N*=3), hypericin (*N*=3) and control/photoactivation groups (*N*=3) are similar in the relative intensity of Ki-67 immunoreactivity. Hypericin/photoactivation group (*N*=3) showed a significant reduction in the relative intensity of Ki-67 immunoreactivity compared with the other three groups. **P<0.01 compared with control, control/photoactivation and hypericin. Bar=20 μm.

**Figure 4:**
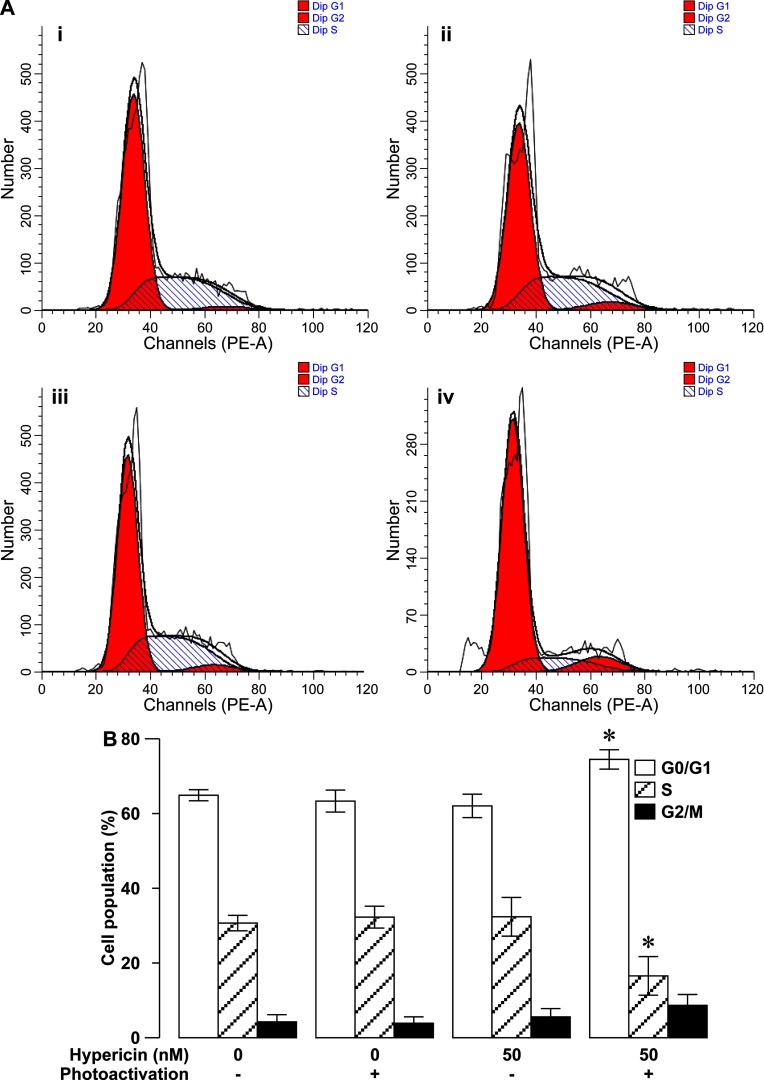
Photoactivated hypericin-induced cell-cycle arrest in the G_0_/G_1_-phase in RINm5F insulinoma cells (**A**) Representative cell-cycle profiles observed in solvent control treatment (control) (**i**), solvent control treatment followed by photoactivation (control/photoactivation) (**ii**), hypericin loading (hypericin) (**iii**) and hypericin loading followed by photoactivation (hypericin/photoactivation) (**iv**) using flow cytometry. (**B**) Flow cytometric quantification of the percentage of cells subjected to control, control/photoactivation, hypericin and hypericin/photoactivation treatments respectively, in the G_0_/G_1_, S and G_2_/M phases of the cell cycle. The percentage of G_0_/G_1_ phase-arrested cells was significantly more in hypericin/photoactivation group (*N*=3) than in the other three groups (*N*=3), whereas the percentage of S-phase cells was significantly less in hypericin/photoactivation group (*N*=3) than in the other three groups (*N*=3). *P<0.05 compared with control, control/photoactivation and hypericin.

Decreases in the viability of rapidly-multiplying cells result from cell proliferation arrest, cell death or both. The inhibitory effect of photoactivated hypericin on the viability of RINm5F insulinoma cells should be attributed to these two cellular events. To determine if one or both of them occurred in our case, we examined the proliferation of RINm5F insulinoma cells loaded with 50 nM hypericin followed by 10-min photoactivation by using Ki-67 immunocytochemistry in combination with DAPI nuclear staining. Ki-67 immunocytochemistry revealed that most of solvent-treated control cells in the absence of photoactivation (Figure 3Aii) and cells loaded with hypericin without photoactivation (Figure 3Av) exhibited moderately intense Ki-67 immunoreactivity. Likewise, the majority of cells subjected to solvent treatment as control followed by photoactivation (control/photoactivation; Figure 3Aviii) also showed a similar intensity of Ki-67 immunoreactivity. In striking contrast, only a small proportion of cells exposed to hypericin followed by photoactivation (hypericin/photoactivation; Figure 3Axi) displayed relatively weak Ki-67 immunoreactivity. Furthermore, DAPI staining showed that cell nuclei were well counterstained with DAPI (Figure 3Ai, iv, vii and x). The overlay of Ki-67 immunoreactivity and DAPI staining illustrated that Ki-67 immunoreactivity was exclusively localized in nuclei exhibiting bright DAPI fluorescence (Figure 3Aiii, vi, ix and xii). Quantification of Ki-67 immunoreactivity demonstrated that there was no significant difference in the relative intensity of Ki-67 immunoreactivity between control, hypericin and control/photoactivation groups (*N*=3, *P*>0.05; Figure 3B). Importantly, the relative intensity of Ki-67 immunoreactivity in hypericin/photoactivation group significantly decreased as compared with the other three groups (*N*=3, *P*<0.01). Furthermore, we also performed flow cytometric cell cycle analysis of cells incubated with 50 nM hypericin followed by 10-min photoactivation. As shown in Figures 4(A) and 4(B), the percentage of G_0_/G_1_-phase-arrested cells significantly increased in hypericin/photoactivation group (*N*=3) as compared with that in control, control/photoactivation and hypericin groups (*N*=3, *P*<0.05). On the contrary, the percentage of S-phase cells significantly decreased in hypericin/photoactivation group (*N*=3) as compared with that in the other three groups (*N*=3, *P*<0.05; Figure 4). There was no significant difference in the percentage of cells in G_0_/G_1_-, S- or G_2_/M-phase between control, control/photoactivation and hypericin groups (*P*>0.05; Figure 4). The results verify that photoactivated hypericin potently inhibits RINm5F insulinoma cell proliferation reflected by decreased expression of the proliferation marker Ki-67 and cell-cycle arrest in the G_0_/G_1_-phase.

**Figure 5:**
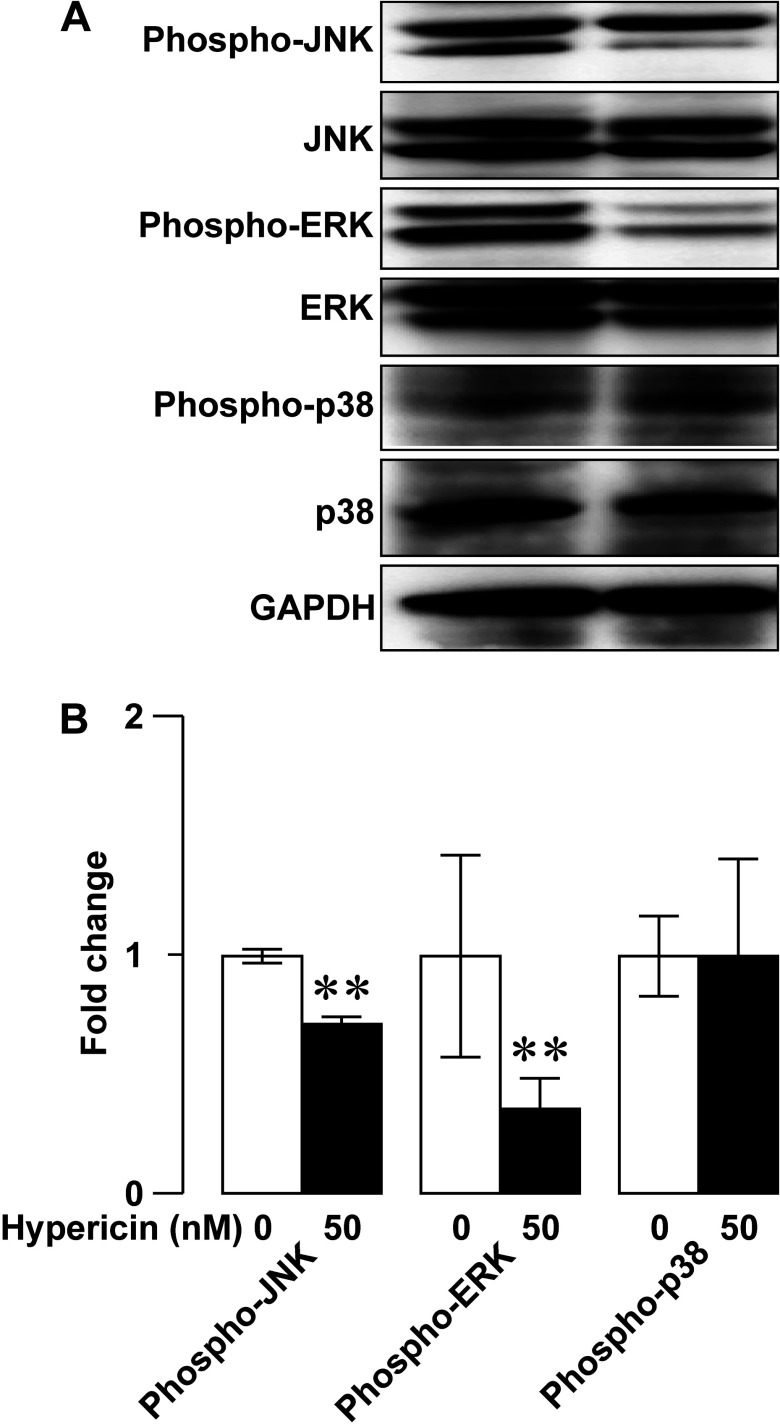
Photoactivated hypericin-induced decrease in the MAPKs phospho-JNK and phospho-ERK in RINm5F insulinoma cells (**A**) Representative immunoblots of cell lysates subjected to solvent control treatment followed by photoactivation (control/photoactivation; left) or hypericin loading followed by photoactivation (hypericin/photoactivation; right). JNK, ERK, phospho-p38, p38 and GAPDH immunoreactive bands in a control/photoactivation-treated sample (left) are similar to corresponding immunoreactive bands in a sample subjected to hypericin/photoactivation (right). Phospho-JNK and phospho-ERK immunoreactive bands in the hypericin/photoactivation-treated sample (right) are weaker than those in the sample subjected to control/photoactivation (left). (**B**) Quantifications of phospho-JNK, phospho-ERK and phosphor-p38 immunoreactive bands in control/photoactivation and hypericin/photoactivation groups. The relative abundance of phospho-JNK and phospho-ERK in hypericin/photoactivation group (*N*=3) significantly decreased as compared with control/photoactivation group (*N*=3). The relative abundance of phospho-p38 in hypericin/photoactivation group did not significantly differ from that in control/photoactivation group (*N*=3). **P<0.01 compared with control/photoactivation.

To dissect mechanistic details of the inhibitory effect of photoactivated hypericin on RINm5F insulinoma cell proliferation, we next evaluated if photoactivated hypericin influenced protein levels of the mitogen-activated protein kinases (MAPKs) phospho-JNK, phospho-ERK and phospho-p38 by employing immunoblot analysis. Anti-phospho-JNK, anti-JNK, anti-phospho-ERK, anti-ERK, anti-phospho-p38, anti-p38 and anti-GAPDH antibodies detected clear phospho-JNK, JNK, phospho-ERK, ERK, phospho-p38, p38 and GAPDH immunoreactive bands respectively (Figure 5A). Solvent-treated control (Figure 5A, left) and hypericin-loaded samples (Figure 5A, right) after photoactivation gave similar intensities of JNK, ERK, phospho-p38, p38 and GAPDH immunoreactive bands, but the hypericin-loaded samples (Figure 5A, right) showed weaker phospho-JNK and phospho-ERK immunoreactive bands than the solvent-treated control samples (Figure 5A, left). Statistical analysis illustrated that photoactivation significantly decreased the relative abundance of phospho-JNK and phosphor-ERK in hypericin group as compared with control group (*N*=3, *P*<0.01) (Figure 5B). However, there is no significant difference in the relative abundance of phospho-p38 between control and hypericin groups (*N*=3, *P*>0.05; Figure 5B). The data verify that photoactivated hypericin down-regulates phospho-JNK and phospho-ERK to inhibit RINm5F insulinoma cell proliferation.

### Photoactivated hypericin induces RINm5F insulinoma cell apoptosis through elevation of cleaved caspase-3, cleaved caspase-9 and the Bax-to-Bcl-2 ratio

**Figure 6:**
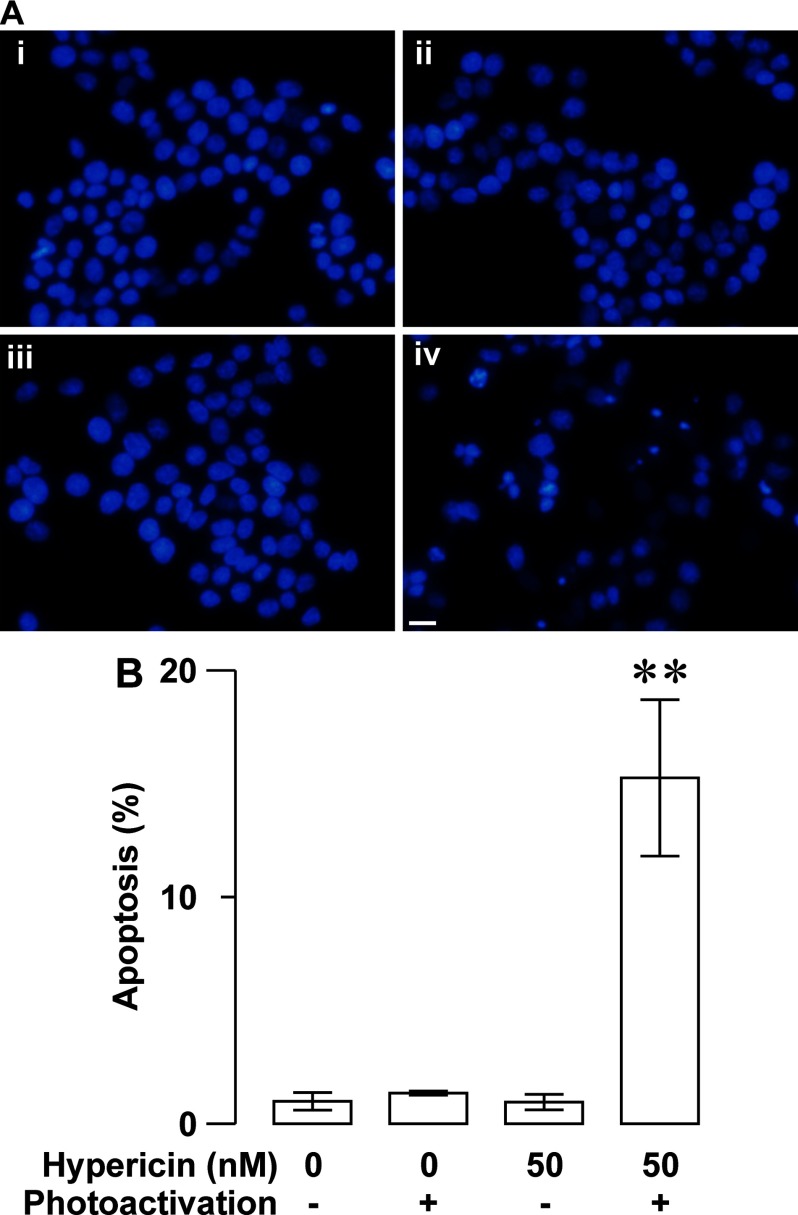
Photoactivated hypericin-induced up-regulation of RINm5F insulinoma cell apoptosis revealed by DAPI-staining analysis (**A**) Sample DAPI fluorescence images were acquired from specimens subjected to solvent control treatment (control), hypericin loading (hypericin), solvent control treatment followed by photoactivation (control/photoactivation) and hypericin loading followed by photoactivation (hypericin/photoactivation) respectively. A hypericin/photoactivation-treated specimen (**iv**) showed several apoptotic profiles characterized by chromatin condensation, nuclear shrinkage and apoptotic body formation, but control- (**i**), hypericin- (**ii**), control/photoactivation-treated specimens (**iii**) rarely displayed apoptotic cells. (**B**) Quantifications of the percentage of apoptotic cells in control, hypericin, control/photoactivation and hypericin/photoactivation groups. The percentage of apoptotic cells is very low in control (*N*=3), hypericin (*N*=3) and control/photoactivation (*N*=3) groups, but significantly increased in hypericin/photoactivation group (*N*=3). **P<0.01 compared with control, control/photoactivation and hypericn. Bar=20 μm.

The photoactivated hypericin-induced reduction in the viability of RINm5F insulinoma cells can also be due to apoptosis triggered by hypericin phototoxicity. Therefore, we measured the apoptotic effect of photoactivated hypericin on RINm5F insulinoma cells by applying DAPI staining. Figure 6(A) shows that apoptotic cells characterized by chromatin condensation, nuclear shrinkage and apoptotic body formation often appeared in hypericin-loaded specimens after photoactivation (Figure 6Aiv). However, apoptotic profiles rarely occurred in solvent-treated control specimens with (control/photoactivation; Figure 6Aiii) and without photoactivation (control; Figure 6Ai) and hypericin-treated specimens in the absence of photoactivation (hypericin; Figure 6Aii). Summary data show that the percentage of apoptotic cells significantly increased in hypericin/photoactivation group (*N*=3) in comparison with control (*N*=3), control/photoactivation (*N*=3), hypericin groups (*N*=3, *P*<0.01; Figure 6B).

 

**Figure 7:**
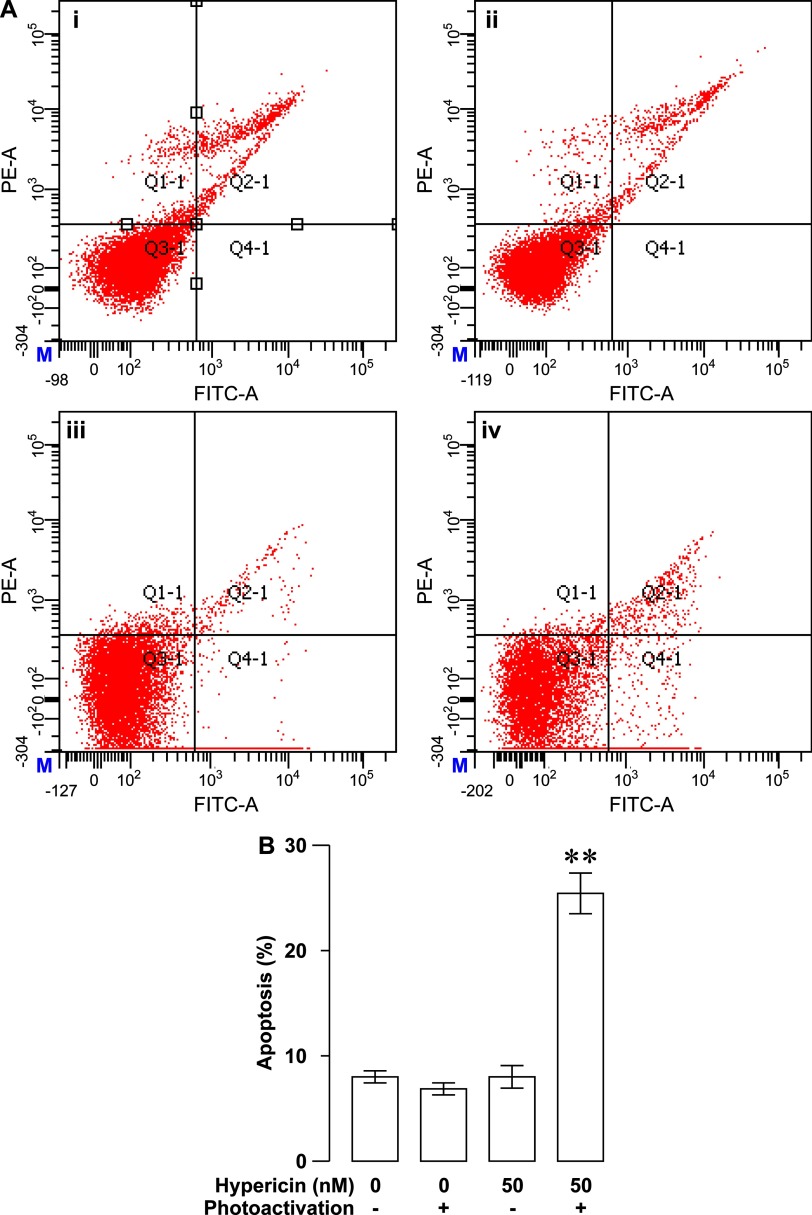
Photoactivated hypericin-induced increase in RINm5F insulinoma cell apoptosis verified by flow cytometric analysis (**A**) Representative flow cytometric dot-plots of cells exposed to solvent control treatment (control) (**i**), solvent control treatment followed by photoactivation (control/photoactivation) (**ii**), hypericin loading (hypericin) (**iii**) and hypericin loading followed by photoactivation (hypericin/photoactivation) (**iv**) respectively. (**B**) Flow cytometric quantification of the percentage of apoptotic cells in control, control/photoactivation, hypericin and hypericin/photoactivation groups. The percentage of apoptotic cells significantly increased in hypericin/photoactivation group (*N*=3) in comparison with the other three groups (*N*=3). **P<0.01 compared with control, control/photoactivation and hypericin.

This apoptotic parameter is very low in the latter three groups. There was no significant difference in the percentage of apoptotic cells between them (*P*>0.05; Figure 6B). Moreover, we also evaluated the apoptotic effect of photoactivated hypericin on RINm5F insulinoma cells by flow cytometry. Flow cytometric analysis of apoptosis showed that the percentage of apoptotic cells was significantly higher in hypericin/photoactivation group (*N*=3) than in control, control/photoactivation and hypericin groups (*N*=3, *P*<0.01; Figures 7A and 7B). The percentage of apoptotic cells in the latter three groups was very low and statistically similar (*P*>0.05; Figures 7A and 7B). It is clear that photoactivated hypericin produce strong phototoxicity to trigger RINm5F insulinoma cell apoptosis.

**Figure 8:**
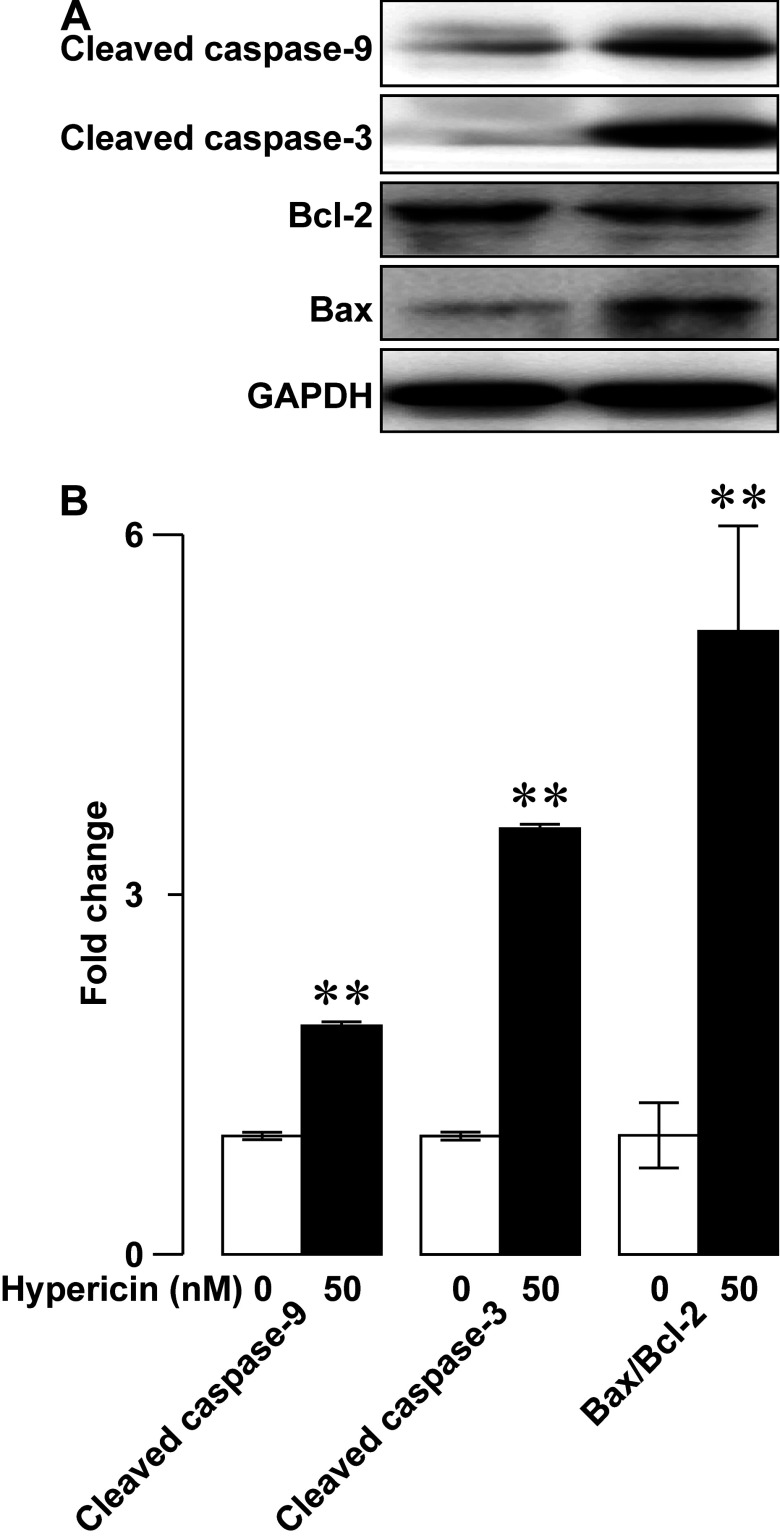
Photoactivated hypericin-induced elevation of the proapoptotic mediators cleaved caspases-3 and cleaved caspases-9 as well as the apoptotic index Bax-to-Bcl-2 ratio in RINm5F insulinoma cells (**A**) Representative immunoblots of cell lysates subjected to solvent control treatment followed by photoactivation (control/photoactivation; left) or hypericin loading followed by photoactivation (hypericin/photoactivation; right). Cleaved caspase-9, cleaved caspase-3 and Bax immunoreactive bands in a sample subjected to hypericin/photoactivation (right) are stronger than those in control/photoactivation-treated sample (left). Bcl-2 immunoreactive band in the hypericin/photoact-treated sample (right) are weaker than that in the sample subjected to control/photoactivation (left). GAPDH immunoreactive band in a control/photoactivation-treated sample (left) resembled that in a sample subjected to hypericin/photoactivation (right). (**B**) Quantifications of cleaved caspase-9, cleaved caspase-3 bands and Bax-to-Bcl-2 ratio in control/photoactivation and hypericin/photoactivation groups. The relative abundance of cleaved caspase-9 and cleaved caspase-3 as well as Bax-to-Bcl-2 ratio in hypericin/photoactivation group (*N*=3) significantly increased as compared with control/photoactivation group (*N*=3). **P<0.01 compared with control/photoactivation.

To mechanistically understand the photoactivated hypericin-induced apoptosis of RINm5F insulinoma cells, we investigated if photoactivated hypericin impinged on the pro-apoptotic mediators cleaved caspases-3, cleaved caspases-9 and the apoptotic index Bax-to-Bcl-2 ratio by using immunoblot analysis. As shown in representative immunoblots, anti-cleaved caspases-3, anti-cleaved caspases-9, anti-Bax, anti-Bcl-2 and anti-GAPDH antibodies correctly recognized cleaved caspases-3, cleaved caspases-9, Bax, Bcl-2 and GAPDH as evidenced by the corresponding immunoreactive bands (Figure 8A). Intensities of immunoreactive bands for cleaved caspases-3, cleaved caspases-9 and Bax in photoactivated samples loaded with hypericin (Figure 8A, right) were stronger than those in samples subjected to the same light exposure but not hypericin loading (Figure 8A, left). The opposite occurred for Bcl-2 immunoreactive bands. Its intensity in the photoactivated samples loaded with hypericin (Figure 8A, right) was weaker than that in the solvent control samples subjected photoactivation (Figure 8A, left). GAPDH immunoreactive bands were similar in both cases (Figure 8A). Quantification of immunoreactive bands revealed that the relative abundance of cleaved caspases-3, cleaved caspases-9 and Bax-to-Bcl-2 ratio significantly increased following photoactivation in hypericin group (*N*=3) in comparison with control group (*N*=3, *P*<0.01; Figure 8B). The results demonstrate that photoactivated hypericin up-regulates cleaved caspases-3, cleaved caspases-9 and Bax-to-Bcl-2 ratio to trigger RINm5F insulinoma cell apoptosis.

## DISCUSSION

The pancreatic β-cell takes centre stage in glucose homoeostasis by operating its unique activity, namely glucose-stimulated insulin secretion, which ensures adequate removal of unnecessary glucose from the blood [34–38]. Excessive insulin secreting cells in insulinomas over-release insulin into the blood and resultant hyperinsulinaemia causes hypoglycaemia leading to a series of severe clinical signs and symptoms including permanent neurological damage and even death [28–30]. This is what exactly happens to insulinoma patients. In a worst-case scenario, malignant insulinoma undergoes distant metastases causing inevitable death in a short period of time [28–30]. Current available therapies cannot satisfactorily treat malignant insulinomas and some benign insulinomas [28–30].

Live-cell confocal fluorescence microscopy has been used to investigate the cellular pharmacokinetics of hypericin in some cancer cell lines such as the human prostate carcinoma cell line DU145, the human nasopharyngeal carcinoma cell lines CNE2 and TW0-1, the human bladder carcinoma cell lines SD andRT112 and the murine keratinocyte cell line PAM-212 [39–42]. Real-time fluorescent imaging reveals that extracellular hypericin is internalized with diverse kinetics in different cell types [39–42]. Hypericin fluorescence peaks at 1 h in DU145 cells and at 2 h in CNE2 and TWO-1 cells as well as SD and RT112 cells, whereas it linearly increases up to 26 h in PAM-212 cells [39–42]. The cellular pharmacokinetics of hypericin in RINm5F insulinoma cells is not known. The present work shows real-time observations of the cellular uptake of hypericin at varying intervals (1, 2, 5, 10 min) in living RINm5F insulinoma cells. Detectable and maximal internalization of hypericin in RINm5F insulinoma cells occurred in 20 and 60 min respectively. This suggests that RINm5F insulinoma cells should be incubated with the photosensitizer hypericin for at least 1 h before photoactivation to gain the maximum photodynamic action of hypericin on the viability of RINm5F insulinoma cells.

In addition, the present observation also demonstrates that hypericin is considerably associated with the plasma membrane, appreciably localized in the sub-plasma membrane region and substantially accumulates in the cytoplasm. Such subcellular localization provides the structural basis for the local phototoxicity of hypericin in these multiple subcellular domains in RINm5F insulinoma cells. In fact, this subcellular localization pattern of hypericin has also been revealed in different cell types [1,19,42–46]. It has been verified that the subcellular localization of hypericin critically underlies hypericin phototoxicity that only occurs in the vicinity of this photosensitizer [1,19,42–46]. This is because the phototoxicity of hypericin relies on the photo-induced reactive oxygen species (ROS) that can only diffuse 20 nm due to their short lifetimes [19]. Photoactivated hypericin produces ROS provoking apoptosis, necrosis and autophagy-related cell death by insulting hypericin-enriched subcellular organelles [1,19,42–46]. It externalizes phosphatidylserine from cell membranes causing loss of plasma membrane integrity and disintegrates the endoplasmic reticulum (ER), Golgi apparatus and mitochondria leading to aberrant Ca^2+^ homoeostasis, cytochrome *c* release and caspase cleavage [1,19,42–46]. Indeed, the present research verifies that photoactivation potently decreases the viability of RINm5F insulinoma cells loaded with hypericin at low nanomolar concentrations. These findings open up a promising prospect for photodynamic therapy against insulinomas.

Importantly, the present work has mechanistically dissected how photoactivated hypericin suppresses the viability of RINm5F insulinoma cells from two different angles, namely cell proliferation and apoptosis. In fact, photoactivation endows hypericin with high anti-proliferative activity against RINm5F insulinoma cells. This is firmly verified by significantly decreased expression of the proliferation marker Ki-67, which appears during all active phases of the cell cycle (G_1_, S, G_2_ and M), but disappears from resting cells (G_0_), in RINm5F insulinoma cells loaded with hypericin followed by photoactivation [47]. In agreement with the analysis of Ki-67 expression, flow cytometric cell-cycle assay demonstrates that photoactivated hypericin arrests RINm5F insulinoma cells in the G_0_/G_1_-phase. This further confirms the anti-proliferative action of photoactivated hypericin in RINm5F insulinoma cells. The MAPK pathway is critical for cell proliferation and often hyperactivated in cancer cells [48]. Furthermore, this pathway has been demonstrated to be a target for photoactivated hypericin in other cell types [1,19]. In the present work, we have examined the major MAPK pathway components JNK, ERK and p38 [48]. Interestingly, photoactivation of hypericin in RINm5F insulinoma cells significantly counteract activation of JNK and ERK, as evidenced by reduction in the phosphorylated forms of JNK and ERK. Taken together, our data demonstrate that photoactivated hypericin interferes with JNK and ERK phosphorylation to arrest RINm5F insulinoma cell proliferation. This underlies, at least in part, the photoactivated hypericin-induced reduction in the viability of RINm5F insulinoma cells.

As aforementioned, we have also investigated the possible involvement of apoptosis in the decreased viability of RINm5F insulinoma cells loaded with hypericin followed by photoactivation. DAPI staining clearly verifies that photoactivation significantly increases apoptotic nuclear profiles appear in hypericin-loaded RINm5F insulinoma cells. Furthermore, flow cytometry demonstrates that photoactivated hyperin effectively drives apoptotic death of RINm5F insulinoma cells. Undoubtedly, photoactivation of hypericin yields substantial phototoxicity to initiate RINm5F insulinoma cell apoptosis. Furthermore, we have found that photoactivated hypericin significantly up-regulates cleaved caspases-9, cleaved caspases-3 and Bax-to-Bcl-2 ratio in RINm5F insulinoma cells. It is most likely that hypericin phototoxicity disintegrates mitochondria by elevating their upstream signal Bax-to-Bcl-2 ratio resulting in cytochrome *c* escape to the cytoplasm where cytochrome *c* interacts with procaspase-9 leading to activation of procaspase-9 by proteolytic cleavage [1,19,49,50]. The cleaved caspase-9 in turn cleaves and activates procaspase-3 [49,50]. Eventually, the cleaved caspase-3 executes apoptosis by cleaving a number of protein substrates that are essential in DNA repair [49,50].

Collectively, the present research lays a solid foundation for implementation of hypericin-mediated photodynamic therapy in treatment of insulinomas, based on the following innovative observations. First, hypericin is efficiently internalized and accumulates in RINm5F insulinoma cells. Second, photoactivated hypericin potently inhibits RINm5F insulinoma cell proliferation through down-regulation of phospho-JNK and phospho-ERK. Finally, photoactivated hypericin effectively provokes RINm5F insulinoma cell apoptosis through activation of caspase-3 and caspase-9 and elevation of the Bax-to-Bcl-2 ratio. Obviously, various hurdles exist in the way of translating the aforementioned observations into clinical application of hypericin-mediated photodynamic therapy in human insulinomas. One of the most difficult hurdles to overcome is to develop a tiny device emitting deep penetrating lights, which can reach insulinomas and their metastatic sites in a minimally invasive way. It is conceivable that LED technology and nanotechnology will help overcome such a hurdle shortly.
